# Profiling the macrofilaricidal effects of flubendazole on adult female *Brugia malayi* using RNAseq

**DOI:** 10.1016/j.ijpddr.2016.09.005

**Published:** 2016-10-01

**Authors:** Maeghan O'Neill, Cristina Ballesteros, Lucienne Tritten, Erica Burkman, Weam I. Zaky, Jianguo Xia, Andrew Moorhead, Steven A. Williams, Timothy G. Geary

**Affiliations:** a Institute of Parasitology, Centre for Host-Parasite Interactions, McGill University, 21,111 Lakeshore Road, Sainte-Anne-de-Bellevue, Quebec H9X 3V9, Canada; b Department of Infectious Diseases, College of Veterinary Medicine, University of Georgia, 501 D.W. Brooks Drive, Athens, GA 30602, USA; c Filariasis Research Reagent Resource Center, Smith College, Northampton, MA 01063, USA; d Department of Biological Sciences, Smith College, Northampton, MA 01063, USA; e Department of Animal Science, McGill University, 21,111 Lakeshore Road, Sainte-Anne-de-Bellevue, Quebec H9X 3V9, Canada

**Keywords:** Filariasis, Macrofilaricide, Benzimidazole, RNAseq, Reproduction, Cuticle, Flubendazole

## Abstract

The use of microfilaricidal drugs for the control of onchocerciasis and lymphatic filariasis (LF) necessitates prolonged yearly dosing. Prospects for elimination or eradication of these diseases would be enhanced by the availability of a macrofilaricidal drug. Flubendazole (FLBZ), a benzimidazole anthelmintic, is an appealing candidate. FLBZ has demonstrated potent macrofilaricidal effects in a number of experimental rodent models and in one human trial. Unfortunately, FLBZ was deemed unsatisfactory for use in mass drug administration campaigns due to its limited oral bioavailability. A new formulation that enables sufficient bioavailability following oral administration could render FLBZ an effective treatment for onchocerciasis and LF. Identification of drug-derived effects is important in ascertaining a dosage regimen which is predicted to be lethal to the parasite *in situ*. In previous histological studies, exposure to FLBZ induced damage to tissues required for reproduction and survival at pharmacologically relevant concentrations. However, more precise and quantitative indices of drug effects are needed. This study assessed drug effects using a transcriptomic approach to confirm effects observed histologically and to identify genes which were differentially expressed in treated adult female *Brugia malayi*. Comparative analysis across different concentrations (1 μM and 5 μM) and durations (48 and 120 h) provided an overview of the processes which are affected by FLBZ exposure. Genes with dysregulated expression were consistent with the reproductive effects observed via histology in our previous studies. This study revealed transcriptional changes in genes involved in embryo development. Additionally, significant downregulation was observed in genes encoding cuticle components, which may reflect changes in developing embryos, the adult worm cuticle or both. These data support the hypothesis that FLBZ acts predominantly on rapidly dividing cells, and provides a basis for selecting molecular markers of drug-induced damage which may be of use in predicting efficacious FLBZ regimens.

## Highlights

• Genes involved in development and cuticle synthesis were differentially expressed.
• Downregulation of adult cuticle genes suggests an alternate mechanism of action.
• One gene was identified as a possible FLBZ-specific marker of damage.

## 1 Introduction

Infections with filarial parasites that cause lymphatic filariasis (LF) and onchocerciasis can lead to debilitating symptoms and cause great economic loss in endemic countries (Zeldenryk et al., 2011, Tyrell, 2013). Control measures have relied on mass drug administration (MDA) of either ivermectin or diethylcarbamazine in combination with albendazole (ABZ) with the aim of eliminating LF and onchocerciasis as public health problems (Molyneux and Zagaria, 2002, Dunn et al., 2015). These drugs act mainly as microfilaricides in an MDA setting, which necessitates yearly dosing for an extended period of time to achieve elimination or local eradication (Cupp et al., 2011). Additionally, MDA programmes for onchocerciasis within Africa are geographically limited due to severe complications associated with acute killing of *Loa loa* microfilaria (mf) in individuals bearing high parasitemia following treatment with ivermectin (Gardon et al., 1997). The introduction of a safe macrofilaricidal drug into current control programs will greatly enhance the elimination of these infections in a timely manner.

Flubendazole (FLBZ), a benzimidazole (BZ) anthelmintic, is a candidate macrofilaricide for use in onchocerciasis and LF control programs. Initially introduced for treatment of livestock for the control of gastrointestinal (GI) parasitic nematode infections (Bradley et al., 1983), FLBZ was subsequently approved for the same indication in humans (Horton, 1990), for which it is highly efficacious (Yangco et al., 1981, Kan, 1983). FLBZ has exhibited very high macrofilaricidal efficacy when administered parenterally in experimental filariasis models (Denham et al., 1979, Mak, 1981, Mackenzie and Geary, 2011) and in a human trial in onchocerciasis (Dominguez-Vazquez et al., 1983). Although available formulations of the drug afford very limited oral bioavailability, recent efforts have been made to re-formulate FLBZ to enable oral dosing (Mackenzie and Geary, 2011, Ceballos et al., 2014, Longo et al., 2014).

Early *in vitro* studies of BZ effects focused on GI nematodes. Ultrastructural observations of *Ascaris suum* 6 h following exposure to mebendazole (Borgers and De Nollin, 1975, Borgers et al., 1975) revealed a loss of microtubule structures in intestinal cells. Further exposure resulted in decreased glycogen content, accumulation of secretory granules near the Golgi and swelling and disruption of microvilli (Borgers and De Nollin, 1975, Borgers et al., 1975, Atkinson et al., 1980). Studies on the exposure of *Toxocara canis* and *A. suum* to FLBZ reported vacuolization of muscle, gonadal tissue, intestine and hypodermis (Hanser et al., 2003). FLBZ-induced damage to reproductive organs of filariae has also been reported (Howells and Delves, 1985, Cárdenas et al., 2010).

Following FLBZ treatment of infected animals, loss of intestinal microtubules from the GI tract of the filarial nematodes *Brugia malayi* and *Litomosoides sigmodontis* was observed when the parasites were recovered as soon as 6 h post-dosing (Franz et al., 1990b). As time after dosing increased, there is an increasing damage to other tissues including the hypodermis and reproductive system.

Definition of the pharmacokinetic profiles needed for efficacy with an orally-bioavailable formulation would be facilitated by knowledge of the time-concentration exposure profiles at which FLBZ is detrimental to the survival of adult filariae. Previous data show that exposure to pharmacologically relevant concentrations of FLBZ elicits damage to the hypodermis, developing embryos, and intestine of adult female *B. malayi*, but this damage is not accompanied by apparent changes in motility or viability (O'Neill et al., 2015). These changes were observed via histology, a method which is challenging for evaluating drug-induced damage. Confirmation of histological damage using a transcriptomic approach can assist in defining target pharmacokinetic profiles for dose selection in advanced development. Determination of FLBZ-specific changes at gene expression level would aide in defining a molecular marker that predicts drug-induced damage.

The present study examines time- and concentration-dependent transcriptomic changes induced in female *B. malayi* after exposure to FLBZ *in vitro.*

## 2 Materials and methods

### 2.1 Parasites

Adult female *B. malayi* were isolated from the peritoneal cavity of jirds (*Meriones unguiculatus*) > 90 days post-infection as described (Moreno and Geary, 2008). Parasites were supplied by the NIH-NIAID Filariasis Research Reagent Resource Center (FR3) at the University of Georgia (UGA), Athens, GA. All animal protocols were reviewed and approved by the UGA Institutional Animal Care and Use Committee, and complied with U.S. Department of Agriculture's regulations (USDA Assurance No. A3437-01).

### 2.2 Experimental design

At UGA, adult female worms were pooled from three individual jirds and randomly distributed among 21 treatment groups (Table 1), ensuring each treatment included three independent replicates with 10 worms per replicate (3 × 10 worms per time point and drug concentration). Worms were washed in RPMI-1640 (BioWhittaker^®^ Classic Cell Culture Media, VWR, Mississauga, ON) supplemented with 1% v/v gentamycin (gentamycin solution, 10 mg/ml Sigma Aldrich Inc., St. Louis, MO, USA), prior to shipping on heat pads overnight to McGill in 15 mL of the same solution. Upon arrival, worms were allocated to individual culture plate wells containing 6 mL RPMI-1640 (Sigma-Aldrich Corp., St. Louis, MO, USA) supplemented with 10% v/v heat-inactivated fetal bovine serum (Sigma-Aldrich Corp., St. Louis, MO, USA), 5% penicillin/streptomycin (Sigma–Aldrich Corp., St. Louis, MO, USA) and 2% v/v gentamycin (Gibco, Thermo Fisher Scientific Inc., Grand Island, NY, USA) with or without drug. FLBZ (Epichem, Murdoch, WA, Australia) was prepared in 100% DMSO and diluted in media to a final concentration of 0.1% v/v DMSO; control media also contained 0.1% DMSO. Worms were incubated for 2 or 5 days at 37 °C and 5% CO_2_, with daily media changes by replacing 3 mL of appropriate media.

### 2.3 RNA extraction

Total RNA was prepared using a previously described protocol (Ballesteros et al., 2016a) which combines organic extraction using Trizol LS reagent (Ambion, Life Technologies, Burlington, ON) and phase lock gel tubes (5 PRIME, Gaithersburg, MD). RNA was purified and concentrated using columns (RNeasy MinElute Cleanup Kit, Qiagen, Valencia, CA) and treated with DNase (Ambion DNA-free Kit, Life Technologies, Burlington, ON). Samples were shipped on dry ice to the NIH-FR3 (Molecular Division) at Smith College (Northampton, MA) for cDNA library preparation and Illumina sequencing.

### 2.4 cDNA library preparation and RNA sequencing

RNA concentration and purity were measured using the Qubit RNA BR Assay Kit (Life Technologies, Q10210, Burlington, ON) on an Agilent 2100 Bioanalyzer (Santa Clara, CA). mRNA was obtained by Poly (A) magnetic isolation (NEBNext Poly (A) mRNA Magnetic Isolation Module, NEB, Ipswich, MA). The enriched mRNA served as template for cDNA library preparation with the NEBNext^®^ Ultra RNA Library Prep Kit Illumina (NEB, E7530) and NEBNext Multiplex Oligos for Illumina (Dual Index Primers Set 1) (NEB, E7600) following the manufacturer's instructions. Assessment of quality, DNA concentration and product size of the cDNA was performed for each library using a Qubit^®^ 2.0 Fluorometer (Life Technologies, Q32866), Qubit^®^ dsDNA BR assay kit (Life Technologies, Q32850), High Sensitivity DNA Analysis Kit (Agilent, 5067-4626) and Agilent 2100 Bioanalyzer. cDNA libraries were sequenced on an Illumina MiSeq Platform employing a 150 base pair paired-end NGS setting.

### 2.5 Data analysis

#### 2.5.1 RNA sequencing analysis

The Mason-Galaxy platform (http://galaxy.iu.edu) was used to execute the RNAseq analysis. FastQ Groomer (v 1.0.4) was used to convert files to FastQ Sanger format and quality assessed using FastQC (v 0.52). Quality assessment was based on %GC content, Illumina adaptor contamination, and average base quality and content. The GC plot is expected to have a normal distribution at the projected GC, multiple peaks was indicative of contamination with non-mRNA material. Based on Fast QC statistics on base sequence content, sequences were trimmed from the 5′ and 3’ ends using FastQ Quality Trimmer (v 1.0.0). Trim Galore (v 0.2.8.1) was used to remove sequences reported as being contaminated with adaptors.

#### 2.5.2 Sequence alignment and transcript quantification

Sequence reads were aligned to the *B. malayi* reference genome (ftp://ftp.wormbase.org/pub/wormbase/species/b_malayi/sequence/genomic/b_malayi.PRJNA10729.WS243.genomic.fa.gz) using TopHat2 (v 0.6), a spliced read mapper built on the short read aligner Bowtie (Kim et al., 2013). The resulting BAM files were used to obtain RNA sequencing metrics (Table 2) using SAM/BAM Alignment Summary Metrics (v 1.56.0). Aligned reads were enumerated using the HTSeq-count package (v 0.6.1) on the Galaxeast-Galaxy platform (http://www.galaxeast.fr/) (Anders et al., 2015) using the mode parameter set to ‘union’ which counts reads overlapping more than one gene model as ambiguous.

#### 2.5.3 Differential gene expression analysis

Differential gene expression was analyzed in edgeR (v 3.10.5) (Robinson et al., 2010) using the web interface NetworkAnalyst (http://networkanalyst.ca) (Xia et al., 2014, Xia et al., 2015). The trimmed mean of M-values (TMM) normalization method was used to correct for library size and reduce RNA compositional effect (Robinson and Oshlack, 2010). Using the Bayes method based on weighted conditional maximum likelihood, tag-wise dispersion parameters were estimated for each gene to facilitate between-gene comparisons (Robinson and Smyth, 2008). Differential expression analyses were performed by conducting pairwise comparisons between the control and drug-treated groups at each time point. Significant genes were selected using a false discovery rate (FDR) cutoff value of 0.15 based on the Benjamini-Hochberg method (Benjamini and Hochberg, 1995).

#### 2.5.4 Bioinformatics analysis

The Wormbase gene name was used to retrieve the protein coding sequence (**Error! Hyperlink reference not valid.**) (Harris et al., 2003) and the Uniprot accession number (http://www.uniprot.org/). Gene Ontology (GO) terms were obtained from Wormbase and nematode.net (v 4.0; http://nematode.net/NN3_frontpage.cgi) (Wylie et al., 2004, Martin et al., 2009). Statistically over-represented GO terms (*p* < 0.05) in gene lists of given treatment groups were identified using WebGestalt (WEB-based Gene SeT AnaLysis Toolkit) (Zhang et al., 2005) using the UniProt accession number for *Caenorhabditis elegans* orthologues (a minimal E-value of 1*10^−20^) of *B. malayi* genes. RNAi phenotypes associated with the *C. elegans* orthologs were retrieved from Wormbase. Venny (v 2.0.2; http://bioinfogp.cnb.csic.es/tools/venny/) was used to create a Venn diagram of overlapping significantly differentially expressed genes (DEGs) in all treatment groups. R was used to generate a volcano plot of differentially expressed genes.

#### 2.5.5 Validation of gene expression

To validate Illumina sequencing results, 3 genes (Bm3608, Bm3280, and Bm4506) were chosen to analyze gene expression by quantitative polymerase-chain reaction (qPCR) methods. Bm5699 (glyceraldehyde-3-phosphate dehydrogenase, GAPDH) was chosen as an endogenous control and normalizer, as its expression was stable over time and across samples. For each RNA sample, 100 ng total RNA were reverse transcribed using the SuperScript VILO MasterMix (Invitrogen, #11755–050, Life Technologies, Burlington, ON) and diluted 5-fold for qPCR reactions. Real-time PCR was performed in triplicate using specific primers designed using Primer-BLAST (http://www.ncbi.nlm.nih.gov/tools/primer-blast/; Table S3). Assays were carried out in 20 μl-reaction volumes containing 10 μl 2X SYBR Select Master Mix (Life Technologies, #4472908), 200 nM final concentration of forward and reverse primers, and 2 μl cDNA in MicroAmp Fast Optical 96-well plates (Life Technologies, # 4346907). Plates were sealed with optical adhesive film (Life Technologies, #4360954) and run in an ABI 7500 real time PCR system using the following program: 50 °C for 2 min, 95 °C for 2 min, 40 cycles defined as 95 °C for 15 s, 58 °C for 15 s, 72 °C for 1 min, followed by a melt curve. Relative expression in the samples of interest was calculated using the ΔΔCt method (Livak and Schmittgen, 2001) and the correlation between Illumina RNA sequencing and qPCR data analyzed by the Pearson test (*p* < 0.05).

## 3 Results

### 3.1 Transcriptomic quantification

The average number of paired-end reads generated from polyA-tailed mRNA ranged from 1.7 to 2.8 million reads (Table 2). Approximately 80% of the sequenced reads were mapped to the reference genome after removal of low quality alignments, accounts for ∼9000 different transcripts. Sequencing depth varied slightly between the treatment groups; however, it is important to note that there is no obvious trend in the number of transcripts between treated and control groups.

### 3.2 FLBZ-dependent changes in the transcriptome

**Fig. 1:**
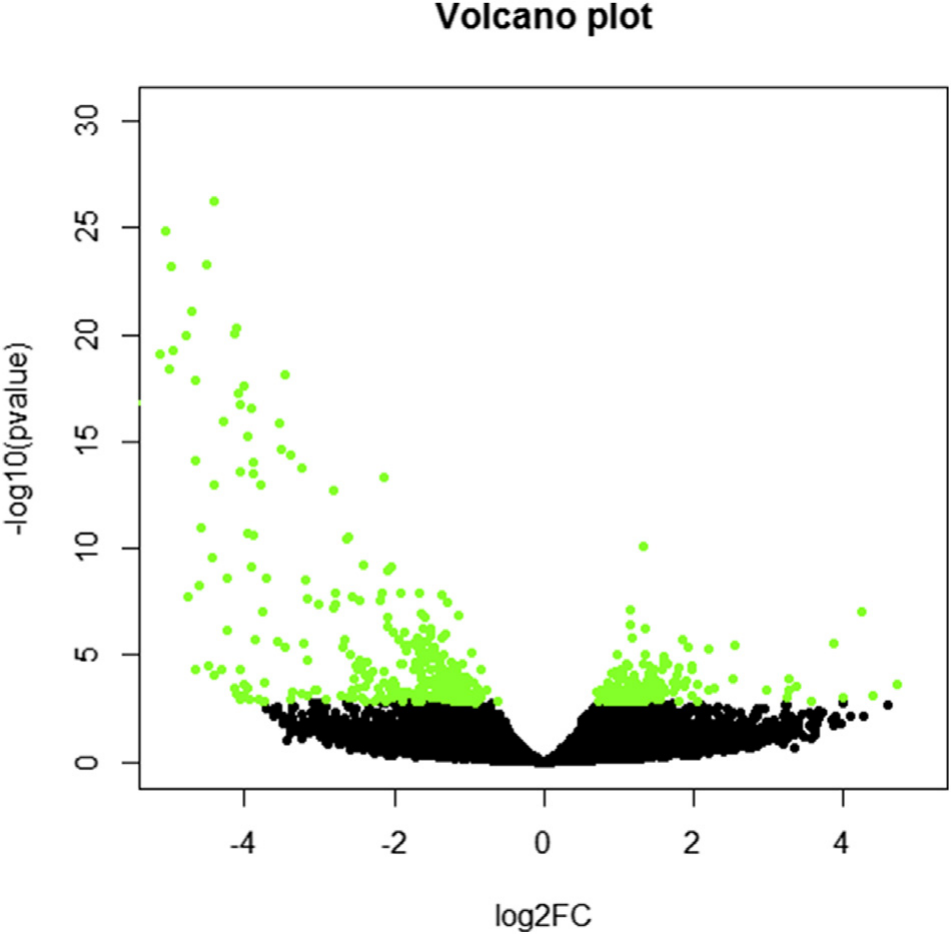
Volcano plot of all differentially expressed genes. Green dots denote significantly differentially expressed genes (FDR<0.15) and black dots symbolize those which are not significant. The x-axis is the fold difference (log 2) between groups and the y-axis represents the log10 of the p-value.

Pairwise comparisons revealed that the number of DEGs ranged from 94 to 159 (Table 3), which accounts for less than 1% of the estimated 14500–17800 protein coding genes in the *B. malayi* genome (Scott and Ghedin, 2009). In general, more genes were downregulated than upregulated (Fig. 1). The largest number of DEGs was found after 120 h exposure to both concentrations of FLBZ. 62.6%–78.6% of DEGs had a known *C. elegans* orthologue.

Exposure to 1 μM FLBZ resulted in 257 genes that were differentially expressed at both time points cumulatively, of which only seven overlapped. These genes were glycosyl hydrolase family protein (Bm4567), clec-1 (Bm3563) PAN domain containing protein (Bm6023), unc-22 (Bm7502), peptidase family M1 containing protein (Bm5654), a putative cuticle collagen (Bm9021) and snf-11 (Bm5517). Exposure to 5 μM FLBZ led to 217 DEGs over both time points with only 5 overlapping genes, including an uncharacterized protein (Bm982), clec-1 (Bm3563), a sugar transporter (Bm5053), oxidoreductase (Bm2014) and snf-11 (Bm5517).

At 48 h, eight genes overlapped between the two concentrations: an uncharacterized protein (Bm8094), two ground-like domain containing proteins (Bm3090, Bm14305), clec-1 (Bm3563), snpn-1 (Bm1903), membrane-anchored cell surface protein (Bm8956), peptidase family M1 containing protein (Bm5654) and snf-11 (Bm5517).

The 120 h time point saw the greatest number of DEGs as well as the greatest number which were shared between the FLBZ concentrations. 82 of the 282 genes overlapped, including genes involved in signaling (2), metabolism (3), transcription (4), transport (7), development (10), collagen or cuticle related (15) or other/unknown functions (41).

**Fig. 2:**
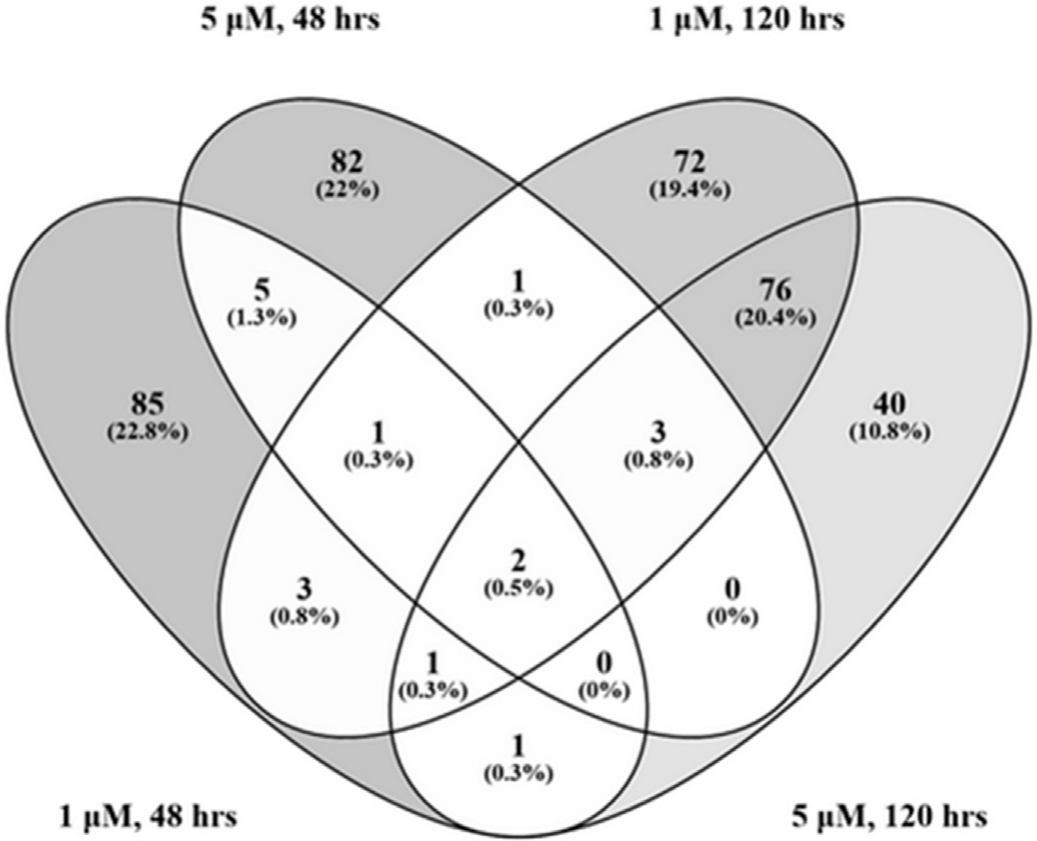
DE genes that overlap among treatment groups. Venn diagram created in Venny 2.0.2.

Only two genes were differentially expressed in all treatment groups (Fig. 2). Bm5517, a sodium-dependent neurotransmitter symporter family protein (snf-11), was upregulated in all treatment groups. An orthologue of clec-1 in *C. elegans* (Bm3563) was also differentially expressed in all treatments. Interestingly, Bm3563 was downregulated in all treatment groups except the 5 μM FLBZ, 120 h group (Fig. 2).

### 3.3 Gene ontology (GO) analysis of differentially expressed genes associated with FLBZ exposure

**Fig. 3:**
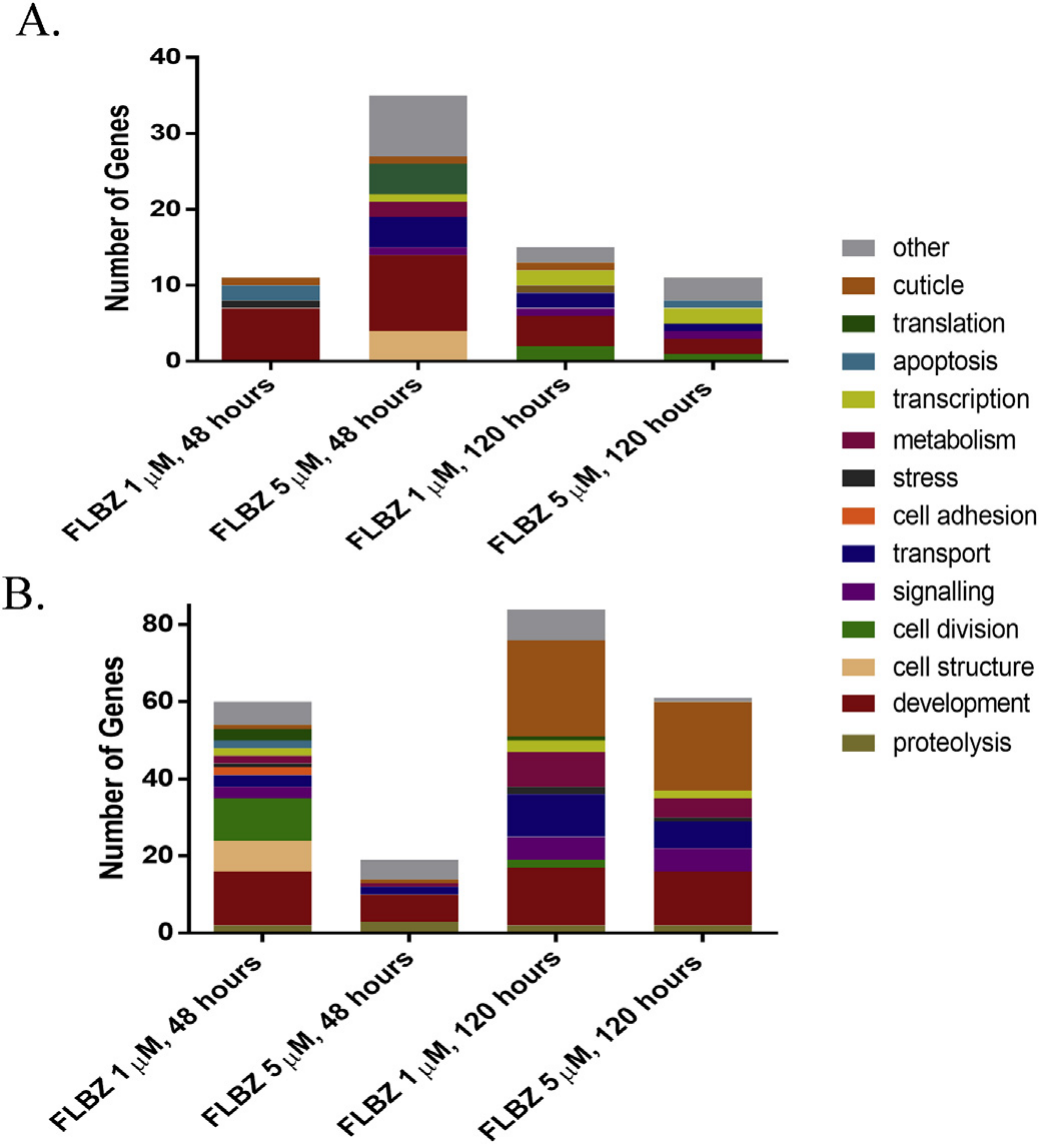
Gene ontology analysis of DE genes associated with FLBZ exposure. Up-regulated (A) and down-regulated (B) genes were manually assigned to GO terms using information available in Wormbase.

To identify the major biological processes affected by FLBZ exposure, GO terms were mined from Wormbase. Because the *B. malayi* genome is not fully annotated, the annotation relies on sequence similarity to evolutionarily related species, with many of the mined GO terms associated with the *C. elegans* orthologues. At all time points and concentrations of FLBZ, the most common GO term for up-regulated genes was “development” (Fig. 3A). Development was also one of the more abundant GO terms for down-regulated genes; however, this changed at the 120 h time point, at which a dramatic increase in the proportion of GO terms relating to the cuticle/collagen was evident (Fig. 3B).

GO term enrichment analysis was conducted with the online interface WebGestalt using *C. elegans* orthologues curated from UniProt. Significantly enriched GO terms varied among the treatment groups. The majority of GO terms for DE genes across treatment groups were related to development and cell division. In the 120 h treatment groups, only two GO terms were enriched at both concentrations: ‘structural constituent of cuticle’ and ‘structural molecule activity’. Interestingly, ‘structural molecule activity’ was the only GO term enriched in all treatment groups.

Genes assigned the GO term ‘structural constituent of cuticle’ were generally down-regulated by FLBZ exposure (Table S1). The only cuticle gene to be up-regulated was Bm5273, a orthologue of *cut*-3 in *C. elegans*, which is required for alae development in larvae (Sapio et al., 2005). Because the cuticle is initially synthesized in late embryogenesis and during each molt (Johnstone, 1994), it is not unexpected to find that the majority of the *C. elegans* orthologues are highly expressed in these developmental stages and for which mutations result in impairment of the cuticle (Table S1). However, 5 of the *C. elegans* orthologues of these 25 genes were most highly expressed in adults (Bm9729, Bm8024, Bm2854, Bm9021, Bm7608), one of which (Bm2854, col-19) is an adult-specific marker for modification and assembly of the cuticle in *C. elegans* (Thein et al., 2003).

Similarly, for genes assigned to “structural molecule activity”, 15 of the 29 genes were collagens (Table S2). Of the five cytoskeletal components assigned this GO term, two were tubulins, including an α-tubulin (Bm9228, *C. elegans* mec-12; Bm10379, *C. elegans* tba-5) and a β-tubulin (Bm4733, *C. elegans* tbb-1). In addition, 6 DEGs are structural components of the ribosome.

### 3.4 qRT-PCR validation

**Fig. 4:**
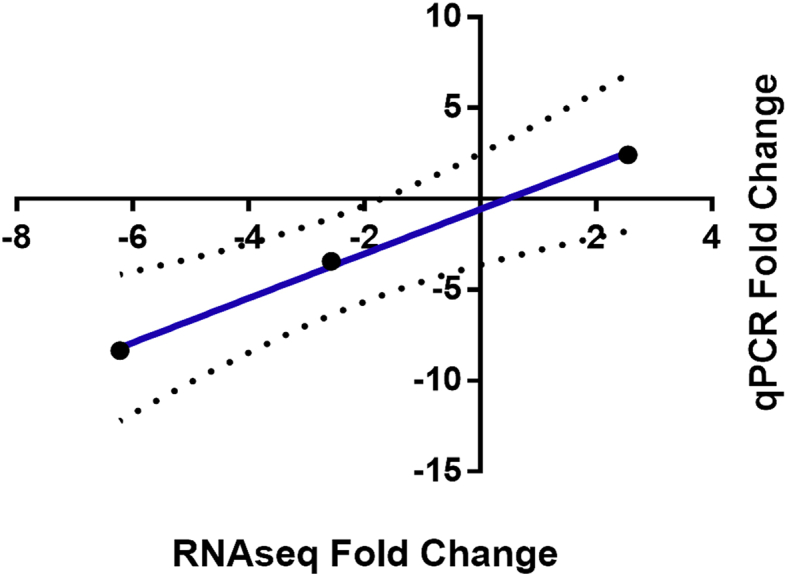
Correlation between qPCR and RNAseq data. The correlation coefficient between RNAseq (x-axis) and qPCR (y-axis) data (log2 fold-change) analyzed by the Pearson test was 0.9978 with a statistical significance p < 0.05.

qRT-PCR was performed to validate changes in the level of gene expression measured by RNAseq. Three significantly up or downregulated genes were compared to the corresponding controls. A robust correlation was found between Illumina RNA sequencing and qPCR data, with a correlation coefficient r = 0.9978 (Fig. 4), analyzed by the Pearson test (*p* < 0.05).

## 4 Discussion

The present study capitalizes on the precision of RNAseq technology for measuring transcript abundance (Wang et al., 2009) to understand transcriptomic changes induced by FLBZ exposure in adult female *B. malayi*. Our results indicate that differentially expressed genes resulting from FLBZ exposure are involved predominantly in structural molecule activity, cuticle, embryogenesis and larval development (Table 4).

### 4.1 Tubulin-related genes

Analysis of significantly enriched GO terms showed that the ‘structural molecule activity’ is the only GO term to be enriched across both FLBZ concentrations at both time points. This was not completely unexpected, as FLBZ, a benzimidazole, is known to inhibit the polymerization of tubulin, an important structural molecule. In fact, three of the 29 genes with structural molecule activity were tubulins, including two α-tubulins (Bm10379, Bm9228) and one β-tubulin (Bm4733). All three genes were up-regulated at various time points, but not consistently across treatment groups (Table S2). It is surprising that FLBZ would elicit an up-regulation of functionally redundant tubulin genes, given that destabilizing drugs typically decrease tubulin synthesis; however, the theory that tubulin monomers auto-regulate transcription (Cleveland, 1988) may suggests that the increasing monomer pool fails to negatively regulate tubulin synthesis, leading instead to increased tubulin synthesis.

It is also interesting to note that the expression of other tubulin-related genes is also altered; *Bma-hcp-6* (log_2_FC of −1.43) the heavy chain of dynein (*Bm-dhc-1*; log_2_FC of −1.14), the dynein intermediate accessory chain (*Bm-dyci-1*,log_2_FC = 2). Differential expression of tubulin genes provides a proof-of-concept of the mechanism of action of FLBZ.

### 4.2 FLBZ dysregulates expression of cuticle-associated genes

The nematode cuticle is a collagen-rich extracellular matrix which covers underlying epithelial cells and is required for normal body morphology, movement, and interactions with the external environment (Page and Winter 2003). The cuticle is first synthesized during late embryogenesis (Page et al., 2014). In the current study, 26 DEGs were annotated as cuticle components (Table S1). Five of them overlapped with the set of DEGs detected during culture of *B. malayi*, indicating that these genes may be related to changes associated with *in vitro* culture, as opposed to drug-induced effects (Ballesteros et al., 2016a). Six additional cuticle-related genes overlapped with a study assessing differential gene expression in ivermectin-treated *B. malayi* which was conducted alongside the current study (Ballesteros et al., 2016b); however, in the ivermectin study, these genes were up-regulated, while they were down-regulated by exposure to FLBZ (Table S1), suggesting they are related to FLBZ treatment.

Utilizing gravid female worms introduces a layer of complexity when assessing the consequence of changes in expression of cuticle-related genes. Differential expression of these genes could stem from a general effect on embryogenesis, through which fewer embryos develop to the stage at which the cuticle is first synthesized. Alternatively (or simultaneously), FLBZ may act on the hypodermis of the adult worm, impairing normal turnover of cuticular components. The genes involved in “Structural constituent of cuticle” (GO: 0042302) are overrepresented in both embryonic and somatic tissue expression (Table S1). *C. elegans* orthologues of these genes reveal that the majority exhibit highest expression in embryos and larval stages (Fig. S1) and have RNAi phenotypes such as “larval lethal”, “molt defective” and “dumpy embryos”. While this suggests that FLBZ-induced cuticular changes are primarily relevant in the context of embryonic development, the *C. elegans* orthologues of several *B. malayi* DEGs exhibit high expression either in all stages (*col-107, col-182, dpy-31, T19B10.2*) or in adults (*col-97, col-130, col-19, col-89*, Fig. S1).

A few of the genes found to be expressed in adults deserve more comment. RNAi knockdown of T19B10.2 (Bm5834 orthologue) impairs the ability of the nematode to resist hypertonic stress (Lamitina and Strange, 2005). The zinc-metalloprotease *dpy-31,* essential to embryonic development, is also required for normal cuticle formation and proper body morphology and is primarily expressed in hypodermal cells (Novelli et al., 2004). *col-130* is not well characterized, but is one of the two genes for which expression is predominantly confined to adult worms (Fig. S1). The other gene is *col-19*, an adult-specific marker for collagen assembly (Thein et al., 2003). It is expressed exclusively in the adult cuticle, and RNAi led to structural defects in the cuticle, including disrupted cuticular ridges. The downregulation of these genes suggests that FLBZ exposure elicits negative consequence on the adult cuticle. While no studies demonstrate cuticular damage by FLBZ, a scanning electron microscopy study of *Wuchereria bancrofti* exposed to either DEC or DEC + ABZ found that ABZ exposure results in cuticular damage (Oliveira-Menezes et al., 2007). Using a TUNNEL-based assay, ABZ was found to damage the adult cuticle in the bovine filariid *Setaria cervi* (Nayak et al., 2011). This study also found extensive damage to the hypodermis. Hypodermal damage is a common theme among the benzimidazoles including FLBZ (Lacey, 1988, Franz et al., 1990a, Kumar and Lai, 1998, Hanser et al., 2003, Shalaby et al., 2009, Cárdenas et al., 2010, O'Neill et al., 2015). Previous studies have shown that short-term exposure to FLBZ leads to extensive damage to *B. malayi* hypodermal tissue (O'Neill et al., 2015). Given that components of the nematode cuticle are synthesized and delivered to the surface through the hypodermis (Page and Winter 2003), it is not surprising that hypodermal tissue damage would result in a dampening of cuticle synthesis.

We know relatively little about the normal turnover rate of cuticular components in adult filariae. Early studies reported that adult surfaces appeared to be quite stable with limited protein shedding (Marshall and Howells, 1986, Scott et al., 1988), but later work suggested that small amounts of surface proteins are released, albeit slowly (Devaney, 1988, Kwan–Lim et al., 1989, Maizels et al., 1989). Given that filariae are long-lived parasites, some rate of turnover of cuticle proteins is expected to occur, and the inhibition of this process by FLBZ could lead to slow death of the worm.

Another possible explanation for the involvement of apparently adult-expressed collagen genes is that components incorporated into the cuticle of developing embryos may originate from the female, as is the case for microfilarial sheath proteins (Selkirk et al., 1991, Jiang et al., 2008). Our ability to determine the consequences of FLBZ exposure on the integrity of the adult cuticle is limited given that only female worms were included in this study. Performing a similar study in male worms could provide insight into the impacts FLBZ has on the adult cuticle in the absence of embryogenesis.

### 4.3 FLBZ influences expression of genes related to embryonic/larval development

Impairments to embryogenesis and larval development emerged as common themes in our analysis. Genes critical for embryogenesis were among the most notable functional categories, including structural molecules, cuticle-related genes, and those involved in mitosis and meiosis.

Effects on embryogenesis can be seen as direct effects on various stages of the cell cycles. A number of genes involved in meiotic chromosome condensation and segregation were down regulated by FLBZ exposure; *viln-1* (Bm2146 orthologue), *hcp-6* (Bm8795 orthologue, Log_2_FC −1.57), the dynein heavy chain (Bm589, Log_2_FC −1.14) and an orthologue of MIX-1 (Bm13786, Log_2_FC −1.57), of PQN-85 (Bm6552, Log_2_FC −1.63), rad-50 (Bm5562, Log_2_FC −1.44),.

Interestingly, genes with roles in anatomical structure morphogenesis (GO:0009653) and developmental process (GO: 0032502) have roles in embryonic elongation. Early embryogenesis entails rapid cell proliferation, but little change in the shape of the embryo (Priess and Hirsh, 1986). Approximately mid-way through embryogenesis, the embryo begins to elongate (Priess and Hirsh, 1986, Ding et al., 2004). This drastic change in shape is heavily dependent on hypodermal development, as stretching of this tissue is essential during the elongation process (Ding et al., 2004). In preparation for morphogenesis, epithelial actin filaments and microtubules organize circumferentially (Priess and Hirsh, 1986). NMY-1 (encoded by Bm4244), a non-muscle myosin which was down regulated in this study (Log_2_FC −1.68), forms filamentous structures in proximity to actin and functions as the motor driving actin constriction (Piekny et al., 2003). Microtubules and the embryonic sheath function together to apply uniform pressure to internal cells as actin filaments contract circumferentially (Priess and Hirsh, 1986). The rate of constriction is regulated by *sma-1* (Bm14776 orthologue in *C. elegans*), which was also found to be down-regulated in this study (log_2_FC −1.62). *sma-1* stabilizes actin fibers during elongation by linking them to the embryonic hypodermis (Praitis et al., 2005). Mutations in *sma-1* slow elongation as actin filaments dissociate from the membrane (McKeown et al., 1998). Pressure distributed evenly across the worm creates an internal hydrostatic pressure that has been suggested to drive elongation (Chin-Sang and Chisholm, 2000).

Studies have shown that muscle contractions are also necessary for elongation (Williams and Waterson, 1994). Contraction is transmitted through the hypodermis to the external surface mechanically through trans-epidermal attachments (TEAs) (Ding et al., 2004). In the present study, two genes related to this process were down-regulated by exposure to FLBZ. *Bma-myo-3* (Bm5021, log_2_FC −1.53) encodes a myosin heavy chain necessary for initiating the assembly of thick filaments (Waterston, 1989). Loss-of-function mutations in *C. elegans myo-3* prevent elongation (Waterston, 1989), highlighting the importance of muscle interaction in morphogenesis. The other gene is *C. elegans* orthologue *vab-10* (Bm7639, Log_2_FC −1.36), which encodes a spectraplakin cross-linker of actin and microtubules (Applewhite et al., 2010). It is required for the interaction between TEAs and the circumferential actin bundles (Ding et al., 2004) and is suggested to protect cells from tension that builds up in the epidermis (Bosher et al., 2003).

Down-regulation of genes involved in early embryo morphogenesis may explain the apparent halt of embryogenesis associated with FLBZ exposure (O'Neill et al., 2016). Short-term *in vitro* exposure to FLBZ followed by long-term maintenance *in vivo* resulted in an increase in late morula stage embryos, the stage preceding the onset of elongation. This effect on genes relating to elongation might well be specific to FLBZ; this general trend was not observed in the concurrently run study summarizing the effects of ivermectin on *B. malayi (*Ballesteros et al., 2016b, suggesting it is not simply a general response to a pharmacological stressor.

It should be noted that virtually all FLBZ-affected genes involved in development were down-regulated; only one gene was up-regulated, accounting for ∼2% of DEGs in this functional category. Considering that FLBZ destabilizes microtubules, which are integral to developmental processes, it is not surprising that these processes would be impeded. Conversely, there is an alternative explanation which may be occurring simultaneously. The evolutionary life-history theory predicts that there is a trade-off between reproduction, growth and survival depending on the availability of resources (Zera and Harshman, 2001). It is conceivable that the insult of drug exposure or resulting damage to tissues involved in nutrient acquisition (O'Neill et al., 2015) could stimulate down-regulation of genes not required for immediate survival, such as those involved in reproduction.

The difficulty in assessing drug-induced damage motivated us to search for FLBZ specific markers of damage. Exposure to relatively high but pharmacologically relevant concentrations (10 μM) of FLBZ for 24 h was not lethal and worms were able to recover (O'Neill et al., 2015, O'Neill et al., 2016). These studies used histological techniques to assess tissues damage in an effort to determine a concentration range which may be lethal over this time-period. Duration to lethality is an important detail in defining target pharmacokinetic profiles. Extended exposure and monitoring of viability, using a FLBZ-specific marker as an index of damage, would provide valuable information for defining dosage profiles.

Comparison of DEGs in each treatment group uncovered two genes that were common in all treatment groups - Bm3563 and Bm5517 (Table 1). To determine the suitability of these genes as markers, their expression profiles were compared to results reported in a study on the transcriptomic changes associated with *in vitro* culture of *B. malayi* (Ballesteros et al., 2016a), as well as the study that identified DEGs associated with ivermectin exposure (Ballesteros et al., 2016b). Bm5517, a sodium-dependent GABA transporter (Mullen et al., 2006) which was up-regulated in all groups in the present study, was also up-regulated in both of the previous transcriptomic studies, rendering it an unsuitable marker. Bm3563 was not differentially expressed following exposure to ivermectin (Ballesteros et al., 2016b), but was up-regulated as a result of *in vitro* culture (Ballesteros et al., 2016a). However, FLBZ exposure caused down-regulation of this gene across all treatment groups. The large difference in expression profiles of this gene between the two studies lends support to the use of this gene as a marker of FLBZ-induced damage.

Bm3563 is an orthologue of *C. elegans clec-1*, which encodes a C-type lectin. Parasitic nematodes exploit lectin receptors to evade the host immune response by secreting C-type lectins (Loukas and Maizels, 2000, Vazquez-Mendoza et al., 2013). However, very little is known about *clec-1*. RNAi studies of *clec-1* in *C. elegans* suggest that it is involved in body morphogenesis, larval development and growth (Ceron et al., 2007), events which are commonly impaired by FLBZ. Further studies are needed to explore the utility of this gene as a marker of damage.

## 5 Conclusions

RNAseq is a valuable technology as it enables unbiased and comprehensive gene expression profiling of complex biological systems. In the current study, we used the RNAseq approach to identify significant genes and biological processes that were being affected in *B. malayi* when challenged with FLBZ. Analysis of GO terms highlights the influence FLBZ on filarial embryonic development, consistent with previous findings (O'Neill et al., 2015, O'Neill et al., 2016). Significantly enriched GO terms are commonly associated with RNAi phenotypes of embryo/larval lethality or impairments to overall morphogenesis for *C. elegans* homologues. We also noted changes in cuticular components, for which all DEGs were down regulated. The exact tissue origins where these changes occur are yet unknown; the contribution from the adult cuticle vs. developing embryos is the subject of future work. One DEG (Bm3563) overlapped all treatment groups and emerged as a possible marker of FLBZ-induced damage. Although more work is necessary to confirm the utility of this gene as a marker, it would be highly beneficial to pharmacodynamic experiments if a dependable, FLBZ-specific marker of drug lethality was available.

## Figures and Tables

**Table 1:** Study design. Worms were randomly allocated to one of three treatment groups. At each time point, three groups of 10 worms were washed, flash-frozen and used for RNA extraction.

Treatment group	Time to RNA isolation after arrival	
	2 Days	5 Days
Control (0.1% DMSO)	3 × 10 worms	3 × 10 worms
1 μM Flubendazole	3 × 10 worms	3 × 10 worms
5 μM Flubendazole	3 × 10 worms	3 × 10 worms

**Table 2:** RNA sequencing summary. Picard alignment summary tool was used to summarize the sequencing and mapping of sequences to the *B. malayi* transcriptome.

Treatment group		Average total # of reads	Aligned reads (%)	High quality alignments (%)	# Of transcripts
48 h	Control	2805561	99.9	82.7	9329
	FLBZ 1 μM	1724686	99.9	82.8	8864
	FLBZ 5 μM	2065240	99.9	82.3	9076
120 h	Control	2462188	99.9	81.8	9054
	FLBZ 1 μM	2087268	99.9	81.1	8827
	FLBZ 5 μM	2771021	99.9	81.8	9126

**Table 3:** Summary of differential gene expression analysis.

Time point	Treatment group	Upregulated genes	Downregulated genes	Total # of DEGs	Genes with *C. elegans* orthologue
48 h	FLBZ 1 μM	18	80	98	77
	FLBZ 5 μM	62	32	94	72
120 h	FLBZ 1 μM	24	135	159	115
	FLBZ 5 μM	19	104	123	77

**Table 4:** GO term enrichment. Top biological processes and molecular functions associated with *C. elegans* orthologues of *B. malayi* genes curated using UniProt. Statistically significantly enriched GO terms are reported as p-value.

GO term	GO ID	48 h		120 h	
		FLBZ 1 μM	FLBZ 5 μM	FLBZ 1 μM	FLBZ 5 μM
*Biological Process*					
Anatomical structure development	GO:0048856	6.46E-06			
Multicellular organismal development	GO:0007275	2.94E-05			
Cell cycle process	GO:0022402	7.46E-05			
M phase	GO:0000279	9.28E-05			
Mitotic chromosome condensation	GO:0007076	9.91E-05			
Cell cycle	GO:0007049	0.0001			
Cell cycle phase	GO:0022403	1.00E-04			
Body morphogenesis	GO:0010171	0.0001			0.084
Developmental process	GO:0032502	2.00E-04			
Anatomical structure morphogenesis	GO:0009653	3.00E-04			
Larval development	GO:0002164	0.0012	0.01		
Locomotion	GO:0040011	0.0018			0.0033
Tissue development	GO:0009888	0.0046			0.0087
Regulation of growth rate	GO:0040009				0.0085
Molting cycle	GO:0042303				0.0067
Positive regulation of growth rate	GO:0040010				0.0084
Molting cycle, collagen and cuticulin-based cuticle	GO:0018996				0.0067
Positive regulation of growth	GO:0045927				0.0031
Regulation of growth	GO:0040008				0.0047
Collagen and cuticulin-based cuticle development	GO:0040002				0.0087
*Molecular Function*					
ATP binding	GO:0005524	8.23E-07			
Adenyl ribonucleotide binding	GO:0032559	8.38E-07			
Adenyl nucleotide binding	GO:0030554	8.38E-07			
Motor activity	GO:0003774	6.03E-06			
Purine ribonucleoside triphosphate binding	GO:0035639	1.15E-05			
Purine ribonucleoside binding	GO:0032550	1.17E-05			
Purine ribonucleotide binding	GO:0032555	1.18E-05			
Purine nucleotide binding	GO:0017076	1.18E-05			
Nucleoside binding	GO:0001882	1.24E-05			
Ribonucleoside binding	GO:0032549	1.24E-05			
Structural molecule activity	GO:0005198	1.51E-02	3.95E-05	2.31E-07	1.03E-08
Structural constituent of cuticle	GO:0042302			8.37E-09	2.84E-09

## References

[bib1] Anders S., Pyl P.T., Huber W. (2015). HTSeq—a Python framework to work with high-throughput sequencing data. Bioinformatics.

[bib2] Applewhite D.A., Grode K.D., Keller D., Zadeh A., Slep K.C., Rogers S.L. (2010). The spectraplakin short stop is an actin–microtubule cross-linker that contributes to organization of the microtubule network. Mol. Biol. Cell.

[bib3] Atkinson C., Newsam R.J., Gull K. (1980). Influence of the antimicrotubule agent, mebendazole, on the secretory activity of intestinal cells of *Ascaridia galli*. Protoplasma.

[bib4] Ballesteros C., Tritten L., O'Neill M., Burkman E., Zaky W.I., Xia J., Moorhead A., Williams S.A., Geary T.G. (2016). The effects of ivermectin on *Brugia malay*i females in vitro: a transcriptomic approach. PLoS Negl. Trop. Dis..

[bib5] Ballesteros C., Tritten L., O'Neill M., Burkman E., Zaky W.I., Xia J., Moorhead A., Williams S.A., Geary T.G. (2016). The effect of *in vitro* cultivation on the transcriptome of adult Brugia malayi. PLoS Negl. Trop. Dis..

[bib6] Benjamini Y., Hochberg Y. (1995). Controlling the false discovery rate: a practical and powerful approach to multiple testing. J. R. Stat. Soc. Ser. B Methodol..

[bib7] Borgers M., De Nollin S. (1975). Ultrastructural changes in *Ascaris suum* intestine after mebendazole treatment *in vivo*. J. Parasitol..

[bib8] Borgers M., De Nollin S., De Brabander M., Thienpont D. (1975). Influence of the anthelmintic mebendazole on microtubules and intracellular organelle movement in nematode intestinal cells. Am. J. Vet. Res..

[bib9] Bosher J.M., Hahn B.-S., Legouis R., Sookhareea S., Weimer R.M., Gansmuller A., Chisholm A.D., Rose A.M., Bessereau J.-L., Labouesse M. (2003). The *Caenorhabditis elegans* vab-10 spectraplakin isoforms protect the epidermis against internal and external forces. J. Cell Biol..

[bib10] Bradley R.E., Guerrero J., Becker H.N., Michael B.F., Newcomb K. (1983). Flubendazole: dose range and efficacy studies against common internal parasites of swine. Am. J. Vet. Res..

[bib11] Cárdenas M.Q., Oliveira-Menezes A., Lanfredi R.M. (2010). Effects of albendazole on *Litomosoides chagasfilhoi* (Nematoda: Filarioidea) females *in vivo*. Parasitol. Res..

[bib12] Ceballos L., Mackenzie C.D., Geary T.G., Alvarez L., Lanusse C. (2014). Exploring the potential of flubendazole in filariasis control: evaluation of the systemic exposure for different pharmaceutical preparations. PLoS Negl. Trop. Dis..

[bib13] Ceron J., Rual J., Chandra A., Dupuy D., Vidal M., van den Heuvel S. (2007). Large-scale RNAi screens identify novel genes that interact with the *C. elegans* retinoblastoma pathway as well as splicing-related components with synMuv B activity. BMC Dev. Biol..

[bib14] Chin-Sang I.D., Chisholm A.D. (2000). Form of the worm: genetics of epidermal morphogenesis in *C. elegans*. Trends Genet..

[bib15] Cleveland D.W. (1988). Autoregulated instability of tubulin mRNAS: a novel eukaryotic regulatory mechanism. Trends biochem. Sci..

[bib16] Cupp E.W., Sauerbrey M., Richards F. (2011). Elimination of human onchocerciasis: history of progress and current feasibility using ivermectin (Mectizan®) monotherapy. Acta Trop..

[bib17] Denham D.A., Samad R., Cho S.Y., Suswillo R.R., Skippins S.C. (1979). The anthelmintic effects of flubendazole on *Brugia pahangi*. Trans. R. Soc. Trop. Med. Hyg..

[bib18] Devaney E. (1988). The biochemical and immunochemical characterisation of the 30 kilodalton surface antigen of *Brugia pahangi*. Mol. Biochem. Parasitol..

[bib19] Ding M., Woo W.-M., Chisholm A.D. (2004). The cytoskeleton and epidermal morphogenesis in *C. elegans*. Exp. Cell Res..

[bib21] Dominguez-Vazquez A., Taylor H.R., Greene B.M., Ruvalcaba-Macias A.M., Rivas-Alcala A.R., Murphy R.P., Beltran-Hernandez F. (1983). Comparison of flubendazole and diethylcarbamazine in treatment of onchocerciasis. Lancet.

[bib24] Dunn C., Callahan K., Katabarwa M., Richards F., Hopkins D., Withers P.C., Buyon L.E., McFarland D. (2015). The contribution of onchocerciasis control and elimination programs toward the achievement of the millennium development goals. PLoS Negl. Trop. Dis..

[bib25] Franz M., Zahner H., Benten P. (1990). Fine-structure alterations in female *Brugia malayi* and *Litomosoides carinii* after *in vivo* treatment with flubendazole. Parasitol. Res..

[bib26] Franz M., Zahner H., Benten P. (1990). Fine-structure alterations in female *Brugia malayi* and *Litomosoides carinii* after *in vivo* treatment with flubendazole. Parasitol. Res..

[bib28] Gardon J., Kamgno J., Folefack G., Gordon-Wendel N., Bouchite B., Boussinesq M. (1997). Marked decreases in the *Loa loa* microfilaraemia six and 12 months after a single dose of ivermectin. Trans. R. Soc. Trop. Med. Hyg..

[bib30] Hanser E., Mehlhorn H., Hoeben D., Vlaminck K. (2003). In vitro studies on the effects of flubendazole against *Toxocara canis* and *Ascaris suum*. Parasitol. Res..

[bib32] Harris T.W., Lee R., Schwarz E., Bradnam K., Lawson D., Chen W., Blasier D., Kenny E., Cunningham F., Kishore R., Chan J., Muller H.M., Petcherski A., Thorisson G., Day A., Bieri T., Rogers A., Chen C.K., Spieth J., Sternberg P., Durbin R., Stein L.D. (2003). WormBase: a cross-species database for comparative genomics. Nucl. Acids Res..

[bib35] Horton R.J. (1990). Benzimidazoles in a wormy world. Parasitol. Today.

[bib36] Howells R.E., Delves C.J. (1985). A simple method for the identification of compounds which inhibit tubulin polymerization in filarial worms. Ann. Trop. Med. Parasitol..

[bib37] Jiang D., Li B.-W., Fischer P.U., Weil G.J. (2008). Localization of gender-regulated gene expression in the filarial nematode *Brugia malayi*. Int. J. Parasitol..

[bib38] Johnstone I.L. (1994). The cuticle of the nematode *Caenorhabditis elegans*: a complex collagen structure. BioEssays.

[bib40] Kan S.P. (1983). The anthelmintic effects of flubendazole on *Trichuris trichiura* and *Ascaris lumbricoides*. Trans. R. Soc. Trop. Med. Hyg..

[bib42] Kim D., Pertea G., Trapnell C., Pimentel H., Kelley R., Salzberg S.L. (2013). TopHat2: accurate alignment of transcriptomics in the presence of insertions, deletions and gene fusions. Genome Biol..

[bib44] Kumar D., Lai S.S. (1998). Mebendazole induced changes in sheep nodular worm in *in vitro* studies. Parasitol. Int..

[bib45] Kwan–Lim G.E., Gregory W.F., Selkirk M.E., Partono F., Maizels R.M. (1989). Secreted antigens of filarial nematodes: a survey and characterization of in vitro excreted/secreted products of adult *Brugia malayi*. Parasite Immunol..

[bib46] Lacey E. (1988). The role of the cytoskeletal protein, tubulin, in the mode of action and mechanism of drug resistance to benzimidazoles. Int. J. Parasitol..

[bib47] Lamitina S.T., Strange K. (2005). Transcriptional targets of DAF-16 insulin signaling pathway protect *C. elegans* from extreme hypertonic stress. Am. J. Physiol.-Cell Physiol..

[bib49] Livak K., Schmittgen T. (2001). Analysis of relative gene expression data using real-time quantitative PCR and the 2(-Delta Delta C(T)) Method. Methods.

[bib50] Longo M., Zanoncelli S., Messina M., Scandale I., Mackenzie C., Geary T., Marsh K., Lindley D., Mazué G. (2014). *In vivo* preliminary investigations of the effects of the benzimidazole anthelmintic drug flubendazole on rat embryos and fetuses. Reprod. Toxicol..

[bib51] Loukas A., Maizels R.M. (2000). Helminth C-type lectins and host–parasite interactions. Parasitol. Today.

[bib52] Mackenzie C.D., Geary T.G. (2011). Flubendazole: a candidate macrofilaricide for lymphatic filariasis and onchocerciasis field programs. Exp. Rev. Anti-infect. Ther..

[bib53] Maizels R.M., Gregory W.F., Kwan-Lim G.-E., Selkirk M.E. (1989). Filarial surface antigens: the major 29 kilodalton glycoprotein and a novel 17–200 kilodalton complex from adult *Brugia malayi* parasites. Mol. Biochem. Parasitol..

[bib54] Mak J.W. (1981). Antifilarial activity of mebendazole and flubendazole on *Breinlia booliati*. Trans. Roy. Soc. Trop. Med. Hyg..

[bib55] Marshall E., Howells R.E. (1986). Turnover of the surface proteins of adult and third and fourth stage larval *Brugia pahangi*. Mol. Biochem. Parasitol..

[bib56] Martin J., Abubucker S., Wylie T., Yin Y., Wang Z., Mitreva M. (2009). Nematode.net update 2008: improvements enabling more efficient data mining and comparative nematode genomics. Nucl. Acids Res..

[bib57] McKeown C., Praitis V., Austin J. (1998). sma-1 encodes a βH-spectrin homolog required for *Caenorhabditis elegans* morphogenesis. Develop.

[bib58] Molyneux D.H., Zagaria N. (2002). Lymphatic filariasis elimination: progress in global programme development. Ann. Trop. Med. Parasitol..

[bib59] Moreno Y., Geary T.G. (2008). Stage- and gender-specific proteomic analysis of *Brugia malayi* excretory-secretory products. PLoS Negl. Trop. Dis..

[bib60] Mullen G.P., Mathews E.A., Saxena P., Fields S.D., McManus J.R., Moulder G., Barstead R.J., Quick M.W., Rand J.B. (2006). The *Caenorhabditis elegans* snf-11 gene encodes a sodium-dependent GABA transporter required for clearance of synaptic GABA. Mol. Biol. Cell.

[bib61] Nayak A., Gayen P., Saini P., Maitra S., Sinha Babu S.P. (2011). Albendazole induces apoptosis in adults and microfilariae of *Setaria cervi*. Exp. Parasitol..

[bib62] Novelli J., Ahmed S., Hodgkin J. (2004). Gene interactions in *Caenorhabditis elegans* define DPY-31 as a candidate procollagen C-proteinase and SQT-3/ROL-4 as its predicted major target. Genetics.

[bib63] O'Neill M., Geary J.F., Agnew D.W., Mackenzie C.D., Geary T.G. (2015). *In vitro* flubendazole-induced damage to vital tissues in adult females of the filarial nematode *Brugia malayi*. Int. J. Parasitol.-Drugs Drug Resist.

[bib64] O'Neill M., Mansour A., Dicosty U., Geary J., Dzimianski M., McCall S., McCall J., Mackenzie C., Geary T. (2016). An *in vitro/in vivo* model to analyze the effects of flubendazole exposure on adult female Brugia malayi. PLoS Negl. Trop. Dis..

[bib66] Oliveira-Menezes A., Lins R., Norões J., Dreyer G., Lanfredi R.M. (2007). Comparative analysis of a chemotherapy effect on the cuticular surface of *Wuchereria bancrofti* adult worms *in vivo*. Parasitol. Res..

[bib68] Page A.P., Stepek G., Winter A.D., Pertab D. (2014). Enzymology of the nematode cuticle: a potential drug target?. Int. J. Parasitol.-Drugs Drug Resist.

[bib69] Page A.P., Winter A.D. (2003). Enzymes involved in the biogenesis of the nematode cuticle. Adv. Parasitol..

[bib70] Piekny A.J., Johnson J.-L.F., Cham G.D., Mains P.E. (2003). The *Caenorhabditis elegans* nonmuscle myosin genes nmy-1 and nmy-2 function as redundant components of the let-502/Rho-binding kinase and mel-11/myosin phosphatase pathway during embryonic morphogenesis. Develop.

[bib71] Praitis V., Ciccone E., Austin J. (2005). SMA-1 spectrin has essential roles in epithelial cell sheet morphogenesis in *C. elegans*. Dev. Biol..

[bib72] Priess J.R., Hirsh D.I. (1986). *Caenorhabditis elegans* morphogenesis: the role of the cytoskeleton in elongation of the embryo. Dev. Biol..

[bib73] Robinson M.D., McCarthy D.J., Smyth G.K. (2010). edgeR: a Bioconductor package for differential expression analysis of digital gene expression data. Bioinformatics.

[bib74] Robinson M.D., Oshlack A. (2010). A scaling normalization method for differential expression analysis of RNA-seq data. Genome Biol..

[bib75] Robinson M.D., Smyth G.K. (2008). Small-sample estimation of negative binomial dispersion, with applications to SAGE data. Biostatistics.

[bib77] Sapio M.R., Hilliard M.A., Cermola M., Favre R., Bazzicalupo P. (2005). The Zona Pellucida domain containing proteins, CUT-1, CUT-3 and CUT-5, play essential roles in the development of the larval alae in *Caenorhabditis elegans*. Dev. Biol..

[bib78] Scott A.L., Diala C., Moraga D.A., Ibrahim M.S., Redding L., Tamashiro W.K. (1988). *Dirofilaria immitis*: biochemical and immunological characterization of the surface antigens from adult parasites. Exp. Parasitol..

[bib79] Scott A.L., Ghedin E. (2009). The genome of *Brugia malayi* — All worms are not created equal. Parasitol. Int..

[bib81] Selkirk M.E., Yazdanbakhsh M., Freedman D., Blaxter M., Cookson E., Jenkins R.E., Williams S. (1991). A proline-rich structural protein of the surface sheath of larval *Brugia* filarial nematode parasites. J. Biol. Chem..

[bib82] Shalaby H., Abdel-Shafy S., Abdel-Rahman K., Derbala A. (2009). Comparative in vitro effect of artemether and albendazole on adult *Toxocara canis*. Parasitol. Res..

[bib84] Thein M.C., McCormack G., Winter A.D., Johnstone I.L., Shoemaker C.B., Page A.P. (2003). *Caenorhabditis elegans* exoskeleton collagen COL-19: an adult-specific marker for collagen modification and assembly, and the analysis of organismal morphology. Dev. Dyn..

[bib85] Tyrell E. (2013). Socioeconomic burden of lymphatic filariasis in Georgetown, Guyana. Trop. Med. Int. Health.

[bib87] Vazquez-Mendoza A., Carrero J.C., Rodriguez-Sosa M. (2013). Parasitic infections: a role for C-type lectin receptors. Biomed. Res. Int..

[bib88] Wang Z., Gerstein M., Snyder M. (2009). RNA-Seq: a revolutionary tool for transcriptomics. Nat. Rev. Genet..

[bib89] Waterston R.H. (1989). The minor myosin heavy chain, mhcA, of *Caenorhabditis elegans* is necessary for the initiation of thick filament assembly. EMBO J..

[bib90] Williams B., Waterson R. (1994). Genes critical for muscle development and function in *Caenorhabditis elegans* identified through lethal mutations. J. Cell Biol..

[bib91] Wylie T., Martin J.C., Dante M., Mitreva M.D., Clifton S.W., Chinwalla A., Waterston R.H., Wilson R.K., McCarter J.P. (2004). Nematode.net: a tool for navigating sequences from parasitic and free-living nematodes. Nucl. Acids Res..

[bib92] Xia J., Benner M.J., Hancock R.E. (2014). NetworkAnalyst–integrative approaches for protein-protein interaction network analysis and visual exploration. Nucl. Acids Res..

[bib93] Xia J., Gill E.E., Hancock R.E. (2015). NetworkAnalyst for statistical, visual and network-based meta-analysis of gene expression data. Nat. Protoc..

[bib94] Yangco B.G., Klein T.W., Deresinski S.C., Vickery A.C., Craig C.P. (1981). Flubendazole and mebendazole in the treatment of trichuriasis and other helminthiases. Clin. Ther..

[bib95] Zeldenryk L.M., Gray M., Speare R., Gordon S., Melrose W. (2011). The emerging story of disability associated with lymphatic filarisis: a critical review. PLoS Negl. Trop. Dis..

[bib96] Zera A.J., Harshman L.G. (2001). The physiology of life history trade-offs in animals. Ann. Rev. Ecol. Syst..

[bib97] Zhang B., Kirov S., Snoddy J. (2005). WebGestalt: an integrated system for exploring gene sets in various biological contexts. Nucl. Acids Res..

