# Supplementary material for: Profiling the macrofilaricidal effects of flubendazole on adult female Brugia malayi using RNAseq
Source: Int J Parasitol Drugs Drug Resist. 2016 Oct 1;6(3):288–96. doi: 10.1016/j.ijpddr.2016.09.005 (PMC5196492; doi:10.1016/j.ijpddr.2016.09.005)
Supplement: Supplementary file 1 [file mmc1.docx]

Supplementary Table 1. Differentially expressed genes assigned the GO term ‘structural constituent of cuticle’ (GO:0042302). Fold Change (log_2_) is reported for 120 hour exposure to flubendazole. Data on developmental stage which exhibits the highest expression in *C. elegans* was mined from wormbase. E – embryos, L – larvae, A - Adults

| *B.malayi* Gene | *C.elegans* orthologue | Fold Change (log_2_) | | Stage with highest expression | RNAi phenotype |
| --- | --- | --- | --- | --- | --- |
|  |  | 1 µM FLBZ | 5 µM FLBZ |  |  |
| Bm6587 | Y46G5A.29 | -5.88 |  |  |  |
| Bm4954 | K09E2.1 | -5.31 |  |  |  |
| Bm4334 | cutl-20 | -4.69 |  | E |  |
| Bm8605 | col-104 | -4.04 | -3.88 | L |  |
| Bm11095 | col-107 | -3.97 |  | All | dumpy, locmotion variant |
| Bm4904 | col-182 | -3.77 | -4.19 | All | sterile, exploding through vulva, larval arrest, lethal |
| Bm9729 | col-97 | -3.49 | -3.36 | L_4_, A | dumpy, morphology and locomotion variant |
| Bm9092 | col-48 | -3.20 | -2.59 | L | lethal, molt defect, body vacuole |
| Bm3465 | cut-1/3 | -3.11 | -2.69 | E, D | larval lethal, molt defect |
| Bm2336 | ptr-1 | -3.09 |  | E, L | dumpy, dauer cuticle varient |
| Bm5827 | T19A5.3 | -3.04 |  | E | larval letal, molt defect |
| Bm3273 | cut-1/3 | -2.72 | -4.51 | E, D | protruding vulva |
| Bm7894 |  | -2.39 | -3.58 |  |  |
| Bm2786 | col-14 | -2.38 | -2.79 | L | protruding vulva |
| Bm8024 | col-130 | -1.95 | -1.88 | A | embryonic lethal |
| Bm5922 | cutl-21 | -1.90 |  | E |  |
| Bm5432 | dpy-31 | -1.56 |  | All | embryonic lethal |
| Bm2605 |  | -1.53 |  |  |  |
| Bm2854 | col-19 | -1.52 | -1.22 | A | breaks in alae, multiple alae |
| Bm9021 | col-89 | -1.50 | -1.24 | L, A | dumpy |
| Bm5834 | T19B10.2 | -1.50 | -0.96 | All | larval lethal, molt defect, lethal, shortened lifespan |
| Bm11071 | col-73 | -1.36 |  | L | dumpy |
| Bm6421 | col-109 |  | -1.4 | L | egg laying defective |
| Bm11074 | col-165 | -1.26 | -1.63 | E | dumpy, dauer cuticle variant, pharyngeal morphology varient |
| Bm7608 | col-97 | -1.18 |  | L_4_, A | dumpy, morphology and locomotion variant |
| Bm5273 | cut-3 | 1.17 |  | E | dumpy, missing alae, pharynx morphology variant |

Supplementary Table 2. Differentially expressed genes assigned the GO term ‘structural molecule activity’ (GO:0005198).

| *B. malayi* Gene | *C.elegans* orthologue | Fold Change (log_2_) | | | | Functional Role |
| --- | --- | --- | --- | --- | --- | --- |
|  |  | 48 hours | | 120 hours | |  |
|  |  | 1 µM FLBZ | 5 µM FLBZ | 1 µM FLBZ | 5 µM FLBZ |  |
| Bm4116 | unc-54 | -1.35 |  |  |  | myosin |
| Bm1706 | mrpl-9 | 0.99 |  |  |  | mitochondrial ribosomal protein |
| Bm14055 | M28.5 |  | 1.12 |  |  | non-histone ribosomal protein |
| Bm7366 | mrps-18A | 1.06 |  |  |  | mitochondrial ribosomal protein |
| Bm8439 | col-45 | 1.74 |  |  |  | constituent of cuticle |
| Bm9238 | act-4 |  | 1.02 |  |  | actin |
| Bm7802 | npp-11 |  | -3.49 |  |  | nucleoporin |
| Bm14726 | rpl-36 |  | 1.45 |  |  | ribosomal protein |
| Bm5011 | mrpl-36 |  | 1.47 |  |  | mitochondrial ribosomal protein |
| Bm10379 | tba-5 |  | 1.55 |  |  | alpha tubulin |
| Bm10355 | col-68 |  | 4.15 |  |  | constituent of cuticle |
| Bm11071 | col-73 |  |  | -1.36 |  | constituent of cuticle |
| Bm4904 | col-182 |  |  | -3.77 | -4.19 | constituent of cuticle |
| Bm11095 | col-107 |  |  | -3.97 |  | constituent of cuticle |
| Bm4733 | ben-1 |  |  | 0.71 |  | beta tubulin |
| Bm9729 | col-97 |  |  | -3.49 | -3.36 | constituent of cuticle |
| Bm2605 | col-89 |  |  | -1.53 | -2.17 | constituent of cuticle |
| Bm8043 | col-104 |  |  | -2.40 | -2.58 | constituent of cuticle |
| Bm9092 | col-48 |  |  | -3.19 | -2.59 | constituent of cuticle |
| Bm2786 | col-14 |  |  |  | -2.79 | constituent of cuticle |
| Bm9228 | mec-12 |  |  | 0.97 | 0.962 | alpha tubulin |
| Bm4024 | grl-4 |  |  | -1.98 | -2.23 | hedgehog-like protein |
| Bm4605 | col-34 |  |  |  | -2.57 | constituent of cuticle |
| Bm11125 | mrpl-17 |  |  |  | -2.88 | mitochondrial ribosomal protein |
| Bm11074 | col-165 |  |  | -1.26 | -1.63 | constituent of cuticle |
| Bm6158 | F53B6.4 |  |  | 1.21 | 1.11 | locomotion |
| Bm6421 | col-109 |  |  |  | -1.35 | constituent of cuticle |
| Bm2854 | col-19 |  |  | -1.52 | -1.22 | constituent of cuticle |
| Bm8024 | col-130 |  |  | -1.95 | -1.87 | constituent of cuticle |

Table S1. Parameters of candidate genes selected for qRT-PCR analysis. For the three genes chosen for validations, primers used and amplicon length are given.

| Gene Name | Bm ID | Primer sequences (forward/reverse) | Amplicon length (bp) | Amplification efficiency (%) | *R^2^ |
| --- | --- | --- | --- | --- | --- |
| GAPDH | Bm5699 | TTTCTGCAGAGGGAGGCAAG  TCAGCGGGATCTTTGCTGTT | 85 | 91.09 | 0.986 |
| let-381 | Bm3608 | GCGGTAAACGGCAGGAGAA  GCCATAGCGATGAGAGCAATG | 54 | 94.097 | 0.99 |
| lips-3 | Bm3280 | TTGGGTCATCGTGTGGATACA  GACAAACGCAAAGACCATAATTTG | 63 | 108.71 | 0.997 |
| Cuticle gene | Bm4605 | TCCGCGGTGACATTTAGCA  GTAAATCATTGGAAGTGTGATGCAT | 62 | 119.12 | 0.995 |

*R^2^, coefficient of determination of the slope of the standard curve
